# Mitochondrial Phylogeography Illuminates the Origin of the Extinct Caspian Tiger and Its Relationship to the Amur Tiger

**DOI:** 10.1371/journal.pone.0004125

**Published:** 2009-01-14

**Authors:** Carlos A. Driscoll, Nobuyuki Yamaguchi, Gila Kahila Bar-Gal, Alfred L. Roca, Shujin Luo, David W. Macdonald, Stephen J. O'Brien

**Affiliations:** 1 Wildlife Conservation Research Unit, Department of Zoology, University of Oxford, Tubney, Oxon, United Kingdom; 2 Laboratory of Genomic Diversity, National Cancer Institute, Frederick, Maryland, United States of America; 3 Koret School of Veterinary Medicine, Faculty of Agriculture, Food and Environment Quality Sciences, Hebrew University of Jerusalem, Rehovot, Israel; 4 Laboratory of Genomic Diversity, SAIC-Frederick, NCI-Frederick, Frederick, Maryland, United States of America; 5 Department of Animal Sciences, University of Illinois at Urbana-Champaign, Urbana, Illinois, United States of America; Freie Universitaet Berlin, Germany

## Abstract

The Caspian tiger (*Panthera tigris virgata*) flourished in Central Asian riverine forest systems in a range disjunct from that of other tigers, but was driven to extinction in 1970 prior to a modern molecular evaluation. For over a century naturalists puzzled over the taxonomic validity, placement, and biogeographic origin of this enigmatic animal. Using ancient-DNA (aDNA) methodology, we generated composite mtDNA haplotypes from twenty wild Caspian tigers from throughout their historic range sampled from museum collections. We found that Caspian tigers carry a major mtDNA haplotype differing by only a single nucleotide from the monomorphic haplotype found across all contemporary Amur tigers (*P. t. altaica*). Phylogeographic analysis with extant tiger subspecies suggests that less than 10,000 years ago the Caspian/Amur tiger ancestor colonized Central Asia via the Gansu Corridor (Silk Road) from eastern China then subsequently traversed Siberia eastward to establish the Amur tiger in the Russian Far East. The conservation implications of these findings are far reaching, as the observed genetic depletion characteristic of modern Amur tigers likely reflects these founder migrations and therefore predates human influence. Also, due to their evolutionary propinquity, living Amur tigers offer an appropriate genetic source should reintroductions to the former range of the Caspian tiger be implemented.

## Introduction

Tigers as a species historically ranged across Eurasia from the Sunda Islands, west through the Indian subcontinent to the Indus river and north along the Pacific seaboard to 60° NL and a wide swath of central Asia from the Russian Far East to eastern Turkey [1], [2]. This wide distribution was primarily influenced by environmental changes associated with Pleistocene glaciation events [3]. Commonly known as the Caspian tiger on the basis of its type locality (N. Persia), the historic range of *Panthera tigris virgata* also included Trans-Caucasia and Eastern Anatolia, with the greatest population densities in the riverine tugai forest systems of Central Asia [1], [2], [4]. During the Middle Ages Caspian tigers were resident across the steppes of Ukraine and southern Russia [4]. Between 1920 and 1970, tiger populations throughout Central Asia declined and disappeared for reasons common to tigers elsewhere: hunting, conversion of their limited habitat to cultivation with a concomitant decline in prey, and conflict with livestock [1], [4]–[6]. The Caspian tiger became extinct in February of 1970 when the last survivor was shot in Hakkari province, Turkey [1], [7].

**Figure 1:**
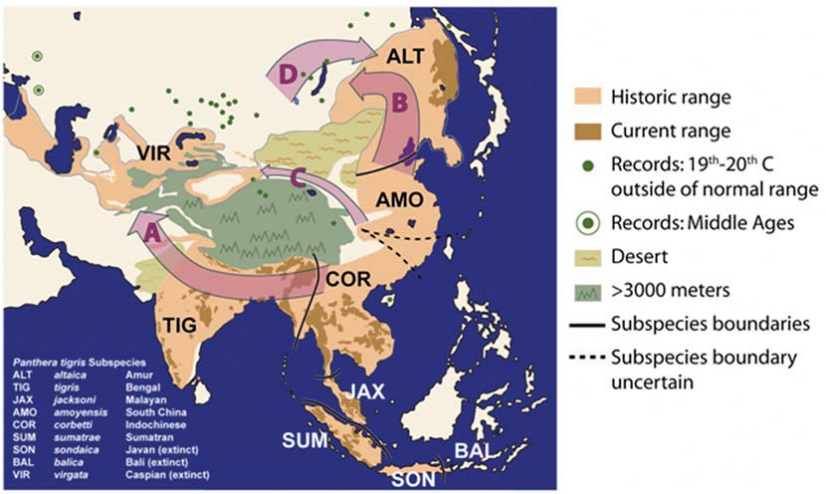
Range of the tiger *Panthera tigris*. Historical range of tiger distribution is shown in light tan and current range is shown in dark tan, while green dots indicate individual historical recordings of tigers outside of normal distribution [1]. Green ‘
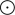
’ indicate records from the Middle Ages [4]. Black lines demarcate presumed subspecies boundaries [3]. Abbreviations correspond to traditionally named tiger subspecies, arranged chronologically by date of naming. 1) *tigris* Linnaeus, 1758[37]; 2) *virgata* Illiger, 1815[38]; 3) *altaica* Temminck, 1844[39]; 4) *sondaica* Temminck, 1844[39]; 5) *amoyensis* Hilzheimer, 1905[40]; 6) *balica* Schwarz, 1912[41]; 7) *sumatrae* Pocock, 1929[42]; 8) *corbetti* Mazak, 1968[43]; 9) *jacksoni* Luo *et al.*, 2004[11]. Lettered arrows indicate postulated dispersal avenues: (A) Indian, southern route; (B) Siberian, northern route; and (C) Silk road/ Gansu route with (D) secondary eastward dispersal. See text for details. Redrawn from Figures 19 and 20 in Mazak [1] and Figure 1 in Kitchener and Dugmore [3].

In the era before molecular taxonomy tiger subspecies definitions were based on classical criteria: geographical origin, gross size and pelage variation (hair length, color, stripe number and patterning) (Figure 1) [3], [6], [8], [9]. Subspecies so described were often spurious as they were sometimes based on a single, possibly aberrant, individual, or from the unknowing sampling of clinal variation [3]. Such methods led to a lack of consensus, repeated taxonomic revision, and debate [10]. Though debate continues, eight tiger subspecies (three of which are extinct) are widely recognized based on these criteria [1], [2], [6]. However the phylogeny of the five extant recognized tiger taxa (*P. t. tigris*, *P. t. altaica*, *P. t. amoyensis*, *P. t. sumatrae*, *P. t. corbetti*) was revisited recently using mitochondrial molecular genetics by Luo *et al.* [11] who affirmed the validity of subspecies ranking for these groups. Additionally, these authors identified an equivalent sub-specific taxon unique to the Malay peninsula south of the Isthmus of Kra, formerly classified within *P. t. corbetti* but now designated as the Malay tiger, *P. t. jacksoni*.

*Panthera tigris virgata* (Illiger, 1815) was the second tiger taxon described following the nominate *Panthera tigris tigris* (Linnaeus, 1758). However, because no holotype specimen of *P. t. virgata* exists, the relative scarcity of specimens, and the unreliability of morphological subspecies-diagnostic characters, the taxonomic validity of *P. t. virgata* has been questioned, its phylogenetic placement relative to other tigers is a matter of speculation, and its biogeographic origin unclear [1], [3], [4], [6]. Here, using well provenanced museum samples and ancient DNA techniques, we explore and interpret the phylogeographic natural history of the Caspian tiger, *P. t. virgata* in the genetic context of the living tiger subspecies, and explore possible routes taken during tiger colonization of Central Asia.

## Results

Twenty (of 23) Caspian tiger museum samples (Table S1) were successfully sequenced for at least one segment of five mitochondrial genes – *ND5*, *ND6*, *CytB*, *ND2*, and *COI* (1257 bp), amplified as eight short amplicons to facilitate PCR of ancient material (see Methods). The amplification targets include 21 single nucleotide polymorphisms (SNPs), of which 14 are diagnostic (fixed differences) for subspecies affiliation in tigers [11], and include four of the four sites diagnostic for *P. t. altaica*, five of the seven for *P. t. amoyensis*, one of the three for *P. t. corbetti*, two of the three for *P. t. tigris* and both sites diagnostic for *P. t. sumatrae*. There are no diagnostic sites for *P. t. jacksoni* though we survey three signature alleles found uniquely in *P.t. jacksoni*.

**Figure 2:**
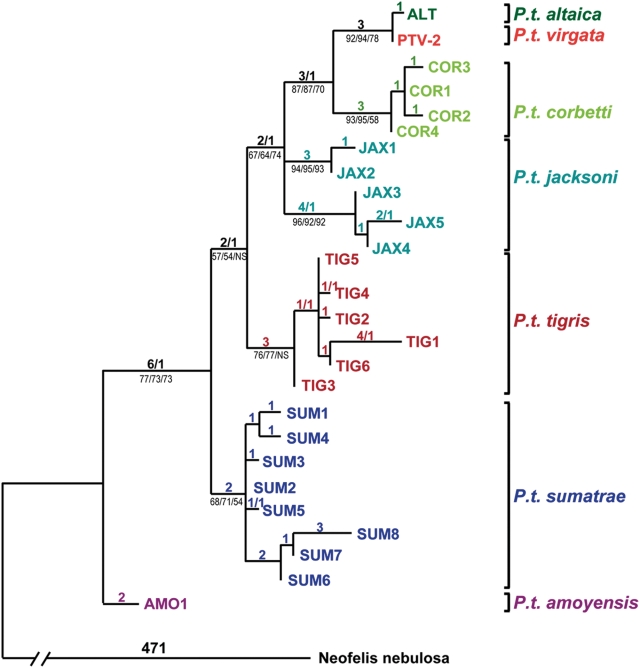
Phylogenetic relationships among tiger mtDNA haplotypes inferred using 4079 bp of concatenated mtDNA sequences (see Table S3). Haplotype designations are color coded by subspecies of the tigers that carried them. PTV-2 is a Caspian tiger (*Panthera tigris virgata*) specimen for which all gene segments attempted (1257 bp) in Caspian tigers were successfully sequenced (see Table S2). Other Caspian tigers produced partial sequences identical to PTV-2. The only exceptions were three individuals, each displaying a single derived nucleotide difference when compared to PTV-2 (found only in that individual and in no other tigers of any subspecies). Likewise, the only mtDNA haplotype carried by Amur or “Siberian” tigers (*P. t. altaica*) proved to be a single derived step away from the haplotype of PTV-2, suggesting a close relationship between the Amur and Caspian tiger subspecies. Tiger haplotypes carried by all but the Caspian subspecies are from a previously published dataset, [11] while a clouded leopard (*Neofelis nebulosa*) sequence (GenBank DQ257669) [14] was used to root the tree. The tree depicted was inferred using maximum parsimony, with the number of steps/homoplasies listed above the branches, while (for major clades) bootstrap percentages are listed below branches for maximum parsimony, maximum likelihood and Neighbour Joining methods. We used full length mtDNA sequences of clouded leopard, leopard and snow leopard to root the tree; all combinations of 1, 2 or 3 outgroups yielded trees with similar topology to the one depicted, with the same basal position for the *P. t. amoyensis* AMO1 haplotype, and a close relationship between *P.t. virgata* and *P. t. altaica* haplotypes.

Seventeen of twenty *P. t. virgata* individuals carried a single distinctive mitochondrial haplotype, while three *P. t. virgata* tigers (Ptv-17, 22, 23) carried autoapomorphic variants (Table 1; Table S2). The amount of mtDNA variability observed in *P. t. virgata* (4 haplotypes/20 individuals), like *P. t. altaica* (1 haplotype/32 individuals), is low relative to other tiger subspecies *P.t. tigris*, (8 haplotypes/19 individuals); *P. t. sumatrae*, (10 haplotypes/31 individuals); *P. t. jacksoni*, (5 haplotypes/28 individuals); *P. t. corbetti* (5 haplotypes/33 individuals) [11]–[13] (Figure 2). Except for Ptv-5, housed in the Moscow Zoo but taken in the wild in Northern Iran, all Caspian tiger specimens are from individuals taken directly from the wild. Because these samples were collected between 1877 and 1951 (i.e., covering ca. 15 tiger generations) from wild tigers in China, Kazakhstan, Afghanistan, and Uzbekistan (see Table S1) it is unlikely they represent the sampling of a single extended family. Moreover, since sample collection took place over the broad geographic range of the subspecies when Central Asian tiger populations were still large, albeit declining, the low endemic mtDNA diversity (relative to other subspecies) indicates that low variability was a natural genetic feature of the 19^th^ century Caspian tiger population and not an anthropogenic effect.

**Table 1:** Tiger (*P. tigris*) mitochondrial haplotypes indicating variable and diagnostic sites.^1^

Taxon	N	# sub-species specific diagnostic sites 3	Gene abbreviation and nucleotide position 2							
			ND5	ND6	CytBa	CytBb	ND2a	ND2b	ND2c	COI
			1	1 1 1 1 1 1	1 1 1	1 1 1	5 5	5 5 5 5	5 5 5	7 7
			3	4 4 4 4 4 4	5 5 5	5 5 5	3 3	5 5 5 6	6 7 7	2 3
			7	5 6 6 6 6 7	5 5 6	6 7 7	3 4	1 1 3 0	7 2 3	8 0
			2	9 1 7 8 8 1	8 9 0	9 4 5	2 9	5 8 3 8	4 8 7	7 4
			2	1 8 9 0 1 1	8 5 2	1 3 6				
*P. t. virgata*	17	0	T	TCCCCA	TGG	CAC	CT	AGGC	TGC	TG
Ptv 22	1		-	TCTCCA	TGG	CAC	- -	AGGC	- - -	- -
Ptv 17	1		T	TCCTCA	TGG	- - -	CT	AGGC	TGC	TG
Ptv 23	1		-	TCCCTA	- - -	CAC	- -	- - - -	- - -	- -
*P. t. altaica*	13	4	|T|	TCCCC|A|	TGG	CAC	|C|T	AGGC	TGC	|C|G
*P. t. amoyensis*	2	5	C	|C|CCCCG	TGG	CAT	T|C|	A|A|GC	|CA|C	TG
*P. t. corbetti*	32	1	C	TCCCCG	T|CA|G	CAC	TT	AGGC	TGC	TG
*P. t. jacksoni* 4	11	0	C	TCCCCG	TGA	TAT	TT	AGGC	TGC	TG
	1		C	TCCCCG	TGG	TAT	TT	AGGC	TGC	TG
	10		C	TCCCCG	TGG	CAT	TT	GGGC	TGC	TG
*P. t. sumatrae*	8	2	C	TCCCCG	TGG	C|G|T	TT	AGG|T|	TGC	TG
	1		C	TCCCCG	CGG	C|G|T	TT	AGG|T|	TGC	TG
	5		C	TCCCCG	TGG	C|G|T	TT	AGG|T|	TGC	TA
*P. t. tigris*	9	2	C	T|T|CCCG	TGG	CAT	TT	AG|A|C	TGC	TG
	6		C	T|T|CCCG	TGG	CAT	TT	AG|A|C	TGT	TG

1 1257 bp sequenced in *P. t. virgata* summarized here, see Table S2 for extended 4 kb haplotypes. Sequence except *P. t. virgata* from [11].

2 Nucleotide positions correspond to the complete *Felis catus* mtDNA sequence [44]. CytB was sequenced from two amplicons, a and b; ND2 was sequenced from three amplicons, a, b, and c.

3 Boxes indicate *P. tigris* subspecies specific diagnostic alleles [11].

4 Underlined are signature alleles (alleles found only within one taxon but not fixed in that taxon). There are no fixed diagnostic alleles in *P. t. jacksoni*, however there are three signature alleles [11]. Data not recovered for an individual indicated by a dash (-).

Aligned sequences define 21 single nucleotide polymorphisms (SNPs), of which 14 are known diagnostic (fixed differences) for subspecies affiliation in extant tigers and seven are subspecies-specific signature alleles which are unique to, but variable within, a given subspecies (Table 1; Table S2). The Caspian tiger sequences differed at all respective diagnostic and signature alleles from *P. t. tigris*, *P. t corbetti*, *P. t. amoyensis*, and *P. t. sumatrae* and matched none of the four *P. t. jacksoni* signature alleles. In contrast, the Caspian haplotype differs from the single *altaica* haplotype only at one of four possible diagnostic sites, a T/C transition at position 7287 in the *COI* gene, where Caspian tigers have the less derived state (T) universal in other tiger subspecies (Table 1).

To place more accurately the Caspian tiger relative to living tiger subspecies we re-assessed the phylogenetic relationships of tiger subspecies using a previously published dataset [11], but here rooted using clouded leopard (*Neofelis nebulosa*) [14], leopard (*Panthera pardus*) [15] and snow leopard (*Panthera uncia*) [16], with the inclusion of Ptv-2, the Caspian tiger for which the longest combined sequence was available (1.26 kb) (see Methods).

The rooting imparted evolutionary polarity to the tiger family tree and showed *P. t. amoyensis* to be basal and *P. t. altaica* to be a sister group to *P. t. corbetti*, while the Caspian tiger haplotype was one step away from that of *P. t. altaica* (Figure 2). The phylogenetic placement and remarkable similarity observed between *P. t. altaica* and *P. t. virgata* indicate that the Amur tiger population is the genetically closest living relative of the extinct Caspian tiger, and strongly implies a very recent common ancestry for the two groups. Russian records from the 19^th^ and early 20^th^ centuries indicate that tigers were sporadically present throughout the region between the core distribution of Caspian and Amur tigers (see Figure 1) and were only hunted out in the modern era [4]. Thus, the actions of industrial-age humans may have been the critical factor in the reciprocal isolation of Caspian and Amur tigers from what was likely a single contiguous population.

The origin of the Amur tiger population is estimated at less than 10,000 years ago by molecular genetic analysis: using a rate of mitochondrial evolution calibrated on the tiger-leopard split (estimated at 2 million ya.), Luo *et al.* [11] inferred that the *P. t. altaica* population, which showed no mtDNA variation, underwent a genetic reduction less than 20,000 ya, that being the time required for a single mutation to appear. The authors then refined their age estimate of the *P. t. altaica* subspecies further to around 10,000 ya. using a standard curve of the relationship of microsatellite allele variance in average repeat size to elapsed time [11]. This estimate is supported by biogeographic reconstructions of tiger range covering the last 20,000 years [3]. Furthermore, paleontological evidence suggests that morphologically modern tigers occurred first around two million years ago in eastern China (in the historic range of modern *P. t. amoyensis*) [8], suggesting that tigers in China may have comprised a stem group that gave rise to modern subspecies. Tigers only recently expanded to the Indian sub-continent (6–12 kya), the Russian Far East (late Pleistocene/Holocene) and Central Asia (Holocene) [1], [3], [4], [10], perhaps impelled by climatic and ecological changes associated with the end of the last glacial period [3]. Therefore, if 19^th^ century Caspian and Amur tigers comprised a single population (as supported by these genetic data), then Caspian tiger diversity (or lack thereof) would likewise date to less than 10,000 years.

## Discussion

The close phylogenetic relationship of the extinct Caspian and the living Amur tigers, plus the unusual reduction in mtDNA diversity of both groups, has important implications for imputing the origins of these tiger subspecies and for modern conservation programs.

Three distinct geographic routes (A–C in Figure 1) have been previously proposed by which tigers might have colonized the Caspian tiger's historic Central Asian range: A) a southern route, via the Indian subcontinent south of the Himalayan plateau [4]; B) a northern route, settling first the Amur region and then traversing Siberia westward, north of the Mongolian steppe [1], [6], [17]; or C) via the historical “Silk Road” through the Gansu corridor, between the Himalayan Plateau and the Mongolian Gobi desert [1].

If colonization had occurred via the Indian subcontinent to the south (route A in Figure 1) a close molecular affinity would exist between Caspian tigers, *P. t. virgata*, and Bengal tigers, *P. t. tigris*, an affinity not supported by this data. Despite the linear proximity between the former ranges of *P. t. virgata* and *P. t. tigris*, significant topographical barriers to dispersal exist. The Tibetan plateau and Himalaya, Hindu Kush, Pamir, and Karakoram ranges are all well above the highest elevation at which tigers have been recorded [1], [6] making transit through these mountains unlikely. Tigers have never been recorded from southern Iran, southern Afghanistan, Baluchistan, or northwestern-most India, presumably because they have difficulty crossing open deserts. The significant mtDNA similarity of *P. t virgata* to living *P. t. altaica*, and their dissimilarity from *P. t. tigris* (Table 1, Figure 2), reflects an extended isolation of *P. t. tigris* from *P. t. altaica* and *P. t. virgata*, effectively ruling out the Southern-route hypothesis.

Of the two remaining hypotheses (B and C in Figure 1), a northern migration route from south China north to Siberia and then west (route B in Figure 1) is unlikely. The almost identical sequences between Amur and Caspian tigers establish their very close relationship. All Amur tigers share a haplotype that is derived from that of the main Caspian haplotype. Although a more complicated history can never be ruled out, the most parsimonious explanation is that the direction of tiger expansion was from west to east. Additionally, the historic ranges of *P. t. corbetti* (Southeast Asia) and *P. t. altaica* (Russian Far East) are not geographically contiguous, the intervening territory having been occupied by *P. t. amoyensis* (central China). A northerly migration (route B) would predict genetic admixture and similarity of *P. t. virgata* with South China tigers *P. t. amoyensis*, consequent of a range overlap of the two subspecies during the postulated migration. However, such genetic similarity is not apparent (Table 1) and *P. t. amoyensis* is clearly distinct as the basal tiger in phylogenetic analyses (Figure 2), reaffirming established morphological distinctiveness [8], [17].

Our phylogenetic inference and the historical geographic range of tigers suggests the Gansu-Silk Road (route C) as the more likely. The present rooted phylogeny corroborates previous maximum likelihood and parsimony analyses [11], and indicate that the Amur tiger (*P. t. altaica*) and the Caspian tiger (*P. t virgata*) are sister taxa to the Indochinese tiger (*P. t. corbetti*) from which they are separated by six mitochondrial steps (five for *P. t. virgata*). Rooting the phylogeny indicates that *P. t. altaica* is the more derived group and *P. t. virgata* the more basal, with *P. t. virgata* differing by a single step from *P. t. altaica*. The presently described *P. t. altaica/ P. t. virgata* affinity with *P. t. corbetti* is consistent with, and may be best explained by, a primary expansion of tigers from China (*P. t. corbetti*) via the Silk Road through the Gansu corridor (route C) directly into the Tarim Basin/Central Asia (*P. t. virgata*) and west towards Anatolia, with a secondary distinctive expansion eastwards (route D) colonizing the historic range of *P. t. altaica* in the Russian Far East.

The central Gansu-Silk Road migration hypothesis (route C in Figure 1) has important implications for interpreting the close similarity and dearth of diversity among 19^th^ century *P. t. virgata* and in modern *P. t. altaica* since behavioral and geographic factors may have interacted to produce mitochondrial invariability in the common ancestor of the two subspecies. Lack of genetic diversity in Caspian tiger samples collected from 1877–1951 (Table 1), well before the 1970 extinction, would suggest that the genetic depletion occurred prior to the early 20^th^ century anthropogenic demographic collapse of this group [12]. The Gansu corridor is defined by mountainous and desert faunal habitats, providing ecological boundaries for a relatively narrow migration corridor (<100 Km). Moreover, tiger behavioral strategies are conducive to restricting gene flow in such a corridor because resident female tigers display strong site fidelity to their territories, which they mark and aggressively defend from immigrants and even their own adult offspring [18]. Because a mother tiger will typically shift her range to make room for a daughter to become established adjacent, such a breeding and land-tenure system could expand a single family matriline mitochondrial haplotype westward through the Gansu, while effectively blocking further migration from the eastern mouth of the corridor. A similar reduction of genetic diversity to a single mtDNA haplogroup was observed among North American pumas due to the re-colonization of North America by pumas from South America through the similarly narrow Isthmus of Panama following the North American mega-faunal extinction in the late Pleistocene around 10,000 years ago [19], [20]. If tiger demographic expansion through the Gansu itself depleted mtDNA diversity in the predecessor of the Amur tiger, then this scenario would displace the current supposition that early 20^th^ century anthropogenic depredation (to as few as 20 Amur tigers) is the predominant cause of genetic depletion in the modern Amur tiger population [12]. This scenario could help explain why the Amur tiger population recovered so well during the 20^th^ century, as deleterious alleles may have been purged prior to its undergoing a recent bottleneck.

### Conclusions

Geographic dispersal of tigers to Central Asia (*P. t. virgata*) and the Russian Far East (*P. t. altaica*) occurred on the order of the last ten thousand years [1], [3], [4], [10], [11]. The ancestral distribution of *P. t. virgata+P. t. altaica* extended from Anatolia to the Russian Far East and this range became discontinuous recently, within the last 200 years, probably through human agency. Prior to this postulated anthropogenic division, these tigers had shared a distinct and united geographic distribution, a unique natural history, and largely concordant phylogenetic characters, the three defining criteria for subspecific taxonomic designation [21] suggesting that *P. t. virgata+P. t. altaica* should be taxonomically considered a single subspecies.

There is debate regarding diagnosability of subspecies based on mtDNA. Nuclear DNA data would help to inform this question. The case for synonymy would be supported if nuclear microsatellite markers that distinguish among other tiger subspecies were found to unite the Caspian and the Amur tiger, although nuclear markers are more difficult to recover with ancient DNA. Additionally, if historical samples of Siberian tigers were found to carry Caspian tiger haplotypes, this would also suggest a lack of differentiation between the two subspecies, strengthening the case for synonymy. A detailed analysis of morphological variation could determine whether diagnosable characters could be found in the remaining sample collections, which are mostly cranial, and the degree of differentiation between the two subspecies. However, because the Caspian tiger was extirpated and few intact specimens are found in museum collections, revisiting the initial morphological assessments is difficult.

Interruption of potential historical gene flow across the ancestral Eurasian distribution of *P. t. altaica+P. t. virgata* may have been too recent (<200 years) to accumulate sub-species level genetic differentiation and a single mtDNA transition may not sufficiently establish the differentiation required to assign each population to separate taxonomic categories. Depending on further study of nuclear genes and morphology, and in view of previous equivocal or conflicting morphological assessments, Caspian and Amur tigers (*P. t. virgata*, Illiger,1815 and *P. t. altaica*, Temminck, 1844, respectively) might be considered as synonymous under the prior *P. t. virgata* trinomial as prescribed by the rules of the ICZN [22], in which case pronouncing the Caspian tiger extinct may have been premature.

Why is the taxonomy of an extinct tiger important? A proper taxonomy is critical to the calculus of species identification and conservation [23], [24]. For example, controversy surrounding the official extinction (in 1987) and molecular taxonomic resurrection (in 1989) of the Florida dusky seaside sparrow was a lesson in the value of an accurate systematic assessment, which should properly allow conservationists to identify those populations of the greatest value and need in order to formulate policy, to disseminate conservation funds, and to manage endangered populations [25]. One potential implication of the present study is that former Caspian tiger habitat in Central Asia is open to reintroductions from Amur stock. As was the case with the dusky seaside sparrow, classical tiger taxonomy has failed to reflect the true phylogenetic distinctions “by giving special emphasis to a presumed biotic partition that was shallow or nonexistent” [26]. The molecular revision, appropriately interpreted, poses a plausible origination scenario for the origins of tiger subspecies with conservation implications for their past, present and future.

## Materials and Methods

Table S1 lists individuals used in this study. Bone fragments or pieces of tissue, about 0.4 cm^2^ were prepared in a physically isolated, ancient DNA laboratory at the National Cancer Institute using appropriate ancient DNA techniques [27]. DNA was extracted from all of the tissues using guanidine thiocyanate (GuHCL) [28] and silica-based purification methods [29]. The extracted DNA was analyzed by PCR [30]. PCR amplification was performed with eight sets of mitochondrial primers (below). Segments were chosen based on previously sequenced regions of other tiger subspecies that were known to be variable and informative. To minimize the possibility of numt amplification in *P. t. virgata*, the primers were designed to avoid regions of known numt in tigers [31]. Furthermore, primers were situated in regions conserved across tiger subspecies. Signs of numt amplification, such as polymorphism in PCR amplicon size or secondary peaks in nucleotide sequences, were not detected. Precautions against numt in the other tiger subspecies had been previously noted [31]; among the outgroups, primers had been used for sequences conserved among the Felidae, while the conservation of open reading frames in the mtDNA genes was an indication that numt had been avoided [14], [16]. Each stage of the procedure (DNA extraction and PCR) was carried out in a dedicated laboratory for ancient DNA studies, which was physically isolated from the Laboratory of Genomic Diversity. The extraction and amplification were carried out using different UV hoods to eliminate contamination of contemporary DNA. All reagents and tubes were cross-linked to prevent contemporary contamination. For the same reason, disposable sterile tubes, filtered tips and sterile reagents and solutions and a dedicated set of pipettes were used throughout the procedure. Multiple negative extraction and amplification controls were included in each PCR reaction to detect contamination. In order to verify the authenticity of the sequences obtained, DNA was sampled and sequenced for every specimen at least twice. The following oligonucleotide primers were designed with PRIMER3 software [32] and were selected such that PCR product length did not exceed 210 bp.

**Table:** 

ND5F	AAACGACGAGCAAGATATTCG
ND5R	ATGCGAGGTTCCGATAATA
ND6-1F	TAACTATACAGTGCTGCAATTCCT
ND6-1R	CTATGGCTACTGAGCCCTACC
CytbaF	TCACCAACCTCCTGTCAGC
CytbaR	GTTATTGGATCCTGTTTCGTGA
CytbbF	CCCTCAGGAATGGTGTCC
CytbbR	GGCGGGGATGTAGTTATCA
ND2aF	GGGGAGTTAACCAAACCGAG
ND2aR	TAGGTTTAAAATTATTATTGTGGGGC
ND2bF	TATCACAAACATGAAACAAAACG
ND2bR	GTATAGGTTAAGTAGTGCTGTTATG
ND2cF	GCCATAACAGCACTACTTAACCTA
ND2cR	TGGGAGTAGTATGGTGGACA
CO1F	GCTGATTGGCCACTCTTCAC
CO1R	ACTCCTATTGACAAGACGTAGTGGA

All PCR reaction were performed using Hi fidelity Taq-Gold (Amersham, Buckinghamshire, UK) to minimize polymerase error in a volume of 25 µl using a touchdown method, starting with a 60°C annealing temperature and ending at 50°C or 48°C. The initial steps were denaturation at 95°C for 10 minutes followed by 45 cycles of 15 seconds at 94°C, 30 seconds of 2 cycles annealing at 60°C, 58°C, 56°C, 54°C, 52°C, and 35 cycles at 50°C or 48°C and 45 seconds elongation at 72°C with final extension of 10 minutes at 72°C. The double stranded PCR products were run on 1.5% low melting agarose gels to determine whether PCR was successful. Positive bands were purified using Microcon 50 (Billerica, MA) and sequenced using the BigDye Terminator system (Applied Biosystems Inc. [ABI], Foster City, CA). Extension products were purified using Sephadex G-50 (Amersham, Buckinghamshire, UK) and resolved on an ABI 3700 or 3730 DNA sequencer. Sequencher 4.5 (Gene Codes Corporation, Ann Arbor, Michigan, USA) software was used to concatenate sequences. Sequences were unambiguously aligned using Clustal-X [33] and visually inspected. Gene identity was established by comparison to homologs in GenBank using BLAST 2.2 [34].

Using clouded leopard, *Neofelis nebulosa*, as an outgroup phylogenetic analyses were performed using maximum parsimony (MP), Neighbor Joining (NJ) and maximum likelihood (ML) methods implemented in PAUP*4.0b10 [35], and employed heuristic searches with 50 replicates of random taxon-addition and TBR branch swapping. The software Modeltest 3.4 [36] was used to determine the model of DNA sequence evolution that best fit the data. For each DNA segment, the model selected was implemented in PAUP*4.0b10 [35] using Modeltest generated likelihood settings for NJ and ML analyses. Bootstrap resampling support was based on 100 (ML) or 2000 (MP, NJ) replicates, with TBR branch swapping of starting trees obtained by stepwise addition. The model of evolution selected by Modeltest corresponded to HKY85+G, with Base = (0.3211 0.2878 0.1384) Nst = 2 TRatio = 22.7043 Rates = gamma Shape = 0.1632 Pinvar = 0. Tree scores were as follows: MP, 4079 total characters, 462 variable sites, 45 parsimony informative, 2 trees found (differing only in relationships among COR6, COR7 and COR8), Length = 531, CI = 0.957, RI = 0.836, RC = 0.800; ML, 1 tree, -Ln likelihood = 7576.77337.

In addition to the clouded leopard, *Neofelis nebulosa* (Nne), full mtDNA sequence became available more recently for two closer outgroup species: the leopard, *Panthera pardus* (Ppa); and the snow leopard, *Panthera uncia* (Pun). To determine whether the choice of outgroup affected tree topology these two outgroups were used in additional phylogenetic analyses. The three outgroups were aligned with tiger sequences and relationships inferred using maximum parsimony, Neighbor Joining and maximum likelihood methods. NJ and ML were run using Modeltest AIC parameters and then, to minimize the effects of the long outgroup, also re-run using Jukes-Cantor. Additionally, the MP tree was inferred for all possible single outgroups and combinations of two outgroups (Nne, Pun, Ppa, Nne+Pun, Nne+Ppa, and Pun+Ppa). In each case, as in the inferences based on the three outgroups, the basal tiger haplotype was *P. t. amoyensis* AMO1 and the close relationship between *P. t. virgata* and *P. t. altaica* was evident in each phylogeny. Thus these results proved quite robust.

To minimize the possibility that long-branch attraction was a factor in the basal position of *P. t. amoyensis*, the data was also rerun with AMO1 and only a few other tiger haplotypes along with the three outgroups. Separate runs examined the position of *P. t. amoyensis* in trees inferred by maximum parsimony using various combinations of tiger haplotypes: (1) AMO1, ALT, COR2, COR8, SUM8, TIG1; (2) AMO1, ALT, SUM8, TIG1; (3) AMO1, SUM8, TIG1. In these trees, terminal branch lengths for other tiger haplotypes were similar to that for *P. t. amoyensis* AMO1. In each case, *P. t. amoyensis* was inferred to have a basal position in the tree. Thus, the basal placement of *P. t. amoyensis* appears to be robust and not due to long-branch attraction.

## Supporting Information

Table S1 Caspian tiger specimens studied
(0.06 MB DOC)

Table S2 P. t. virgata individuals PCR amplified and sequenced at each fragment
(0.05 MB DOC)

Table S3 Variable sites among tigers in an alignment of 4 kb of concatenated mtDNA sequences used in phylogenetic analysis.
(0.22 MB DOC)

## References

[1] Mazák V (1983). Der Tiger: Panthera tigris Linnaeus 1758.

[2] Nowell K, Jackson P, IUCN/SSC Cat Specialist Group (1996). Wild cats : status survey and conservation action plan.

[3] Kitchener AC, Dugmore AJ (2000). Biogeographical change in the tiger, Panthera tigris.. Animal Conservation.

[4] Heptner VG, Sludski AA, Heptner VG, Naumov NP (1972). Mammals of the Soviet Union.. Mammals of the Soviet Union, Carnivora (Hyaenas and Cats).

[5] Sunquist ME, Karanth KU, Sunquist F, Seidensticker J, Jackson P, Christie S (1999). Ecology, behaviour and resiliance of the tiger and its conservation needs.. Riding the tiger: tiger conservation in human-dominated landscapes.

[6] Mazak V (1981). Mammalian Species, Panthera Tigris.. The American Society of Mammalogists.

[7] Can OE (2004). Status, Conservation and Management of Large Carnivores in Turkey. Strasbourg, France: Council of Europe.. T-PVS/Inf (2004) 8 T-PVS/Inf (2004) 8.

[8] Herrington SJ, Tilson RL, Seal US, Minnesota Zoological Garden., IUCN/SSC Captive Breeding Group., IUCN/SSC Cat Specialist Group., editors (1987). Subspecies and the conservation of Panthera tigris: preserving genetic heterogeneity.. Tigers of the world : the biology, biopolitics, management, and conservation of an endangered species.

[9] Seidensticker J, Jackson P, Christie S (1999). Riding the tiger: tiger conservation in human-dominated landscapes.

[10] Kitchener A, Seidensticker J, Christie S, Jackson P (1999). The evolution of the tiger.. Riding the Tiger: Tiger Conservation in human-dominated landscapes.

[11] Luo SJ, Kim JH, Johnson WE, van der Walt J, Martenson J (2004). Phylogeography and genetic ancestry of tigers (Panthera tigris).. PLoS Biol.

[12] Russello M, Gladyshev E, Miquelle D, Caccone A (2004). Potential genetic consequences of a recent bottleneck in the Amur Tiger of the Russian Far East.. Conservation Genetics.

[13] Luo S-J, Johnson WE, Martenson J, Antune A, Martelli P (2008). Subspecies Genetic Assignments of Worldwide Captive Tigers Increase Conservation Value of Captive Populations.. Curr Biol.

[14] Wu XB, Zheng T, Jiang ZG, Wei L (2007). The mitochondrial genome structure of the clouded leopard (Neofelis nebulosa).. Genome.

[15] Wei LaWX.-B (2008).

[16] Wei L, Wu X, Jiang Z (2008). The complete mitochondrial genome structure of snow leopard Panthera uncia.. Mol Biol Rep(Springer Netherlands).

[17] Hemmer H, Tilson RL, Seal US, Minnesota Zoological Garden., IUCN/SSC Captive Breeding Group., IUCN/SSC Cat Specialist Group., editors (1987). The phylogeny of the tiger (Panthera tigris).. Tigers of the world : the biology, biopolitics, management, and conservation of an endangered species.

[18] Smith JLD, McDougal, Charles W, Sunquist, Melvin E, Tilson RL (1987). Female Land Tenure System in Tigers.. Tigers of the World; The Biology, Biopolitics, Management, and Conservation of an Endangered Species.

[19] Culver M, Johnson WE, Pecon-Slattery J, O'Brien SJ (2000). Genomic ancestry of the American puma (Puma concolor).. J Hered.

[20] O'Brien SJ, Johnson WE (2005). Big cat genomics.. Annu Rev Genomics Hum Genet.

[21] O'Brien SJ, Mayr E (1991). Bureaucratic Mischief: Recognizing Endangered Species and Subspecies.. Science.

[22] Ride WDL, International Commission on Zoological Nomenclature.International Trust for Zoological Nomenclature, Natural History Museum (London England), International Union of Biological Sciences. General Assembly (1999). International code of zoological nomenclature = Code international de nomenclature zoologique.

[23] Marris E (2007). Conservation priorities: what to let go.. Nature.

[24] May RM (1990). Taxonomy as destiny.. Nature.

[25] Avise JC, Nelson WS (1989). Molecular Genetic Relationships of the Extinct Dusky Seaside Sparrow.. Science.

[26] Avise JC (2004). Molecular markers, natural history, and evolution.

[27] Cooper A, Poinar HN (2000). Ancient DNA: do it right or not at all.. Science.

[28] Boom R, Sol CJ, Salimans MM, Jansen CL, Wertheim-van Dillen PM (1990). Rapid and simple method for purification of nucleic acids.. J Clin Microbiol.

[29] Hoos M, Paabo S (1993). DNA extraction from Pleistocene bones by silica-based purification method.. Nucleic Acids Res.

[30] Saiki RK, Gelfand DH, Stoffel S, Scharf SJ, Higuchi R (1988). Primer-directed enzymatic amplification of DNA with a thermostable DNA polymerase.. Science.

[31] Kim JH, Antunes A, Luo SJ, Menninger J, Nash WG (2006). Evolutionary analysis of a large mtDNA translocation (numt) into the nuclear genome of the Panthera genus species.. Gene.

[32] Rozen SaS, Helen, Krawetz S, Misener S (2000). Primer3 on the WWW for general users and for biologist programmers.. Bioinformatics Methods and Protocols: Methods in Molecular Biology.

[33] Jeanmougin F, Thompson JD, Gouy M, Higgins DG, Gibson TJ (1998). Multiple sequence alignment with Clustal X.. Trends Biochem Sci.

[34] Altschul SF, Gish W, Miller W, Myers EW, Lipman DJ (1990). Basic local alignment search tool.. J Mol Biol.

[35] Swofford DL (2002). PAUP*: Phylogenetic Analysis Using Parsimony (*and Other Methods). Version 4.0b10.

[36] Posada D, Crandall KA (1998). MODELTEST: testing the model of DNA substitution.. Bioinformatics.

[37] Linnaeus C (1758). Systema naturae per regna tria naturae, 10th edition, volume 1.

[38] Illiger C (1815). Ueberblick der Saugethiere nach ihrer Vertheilung uber die Welttheile..

[39] Temminck C, von Siebold P (1844). Apercu general et specifique sur les mammiferes qui habitent le Japon et les Iles qui en dependant.. Fauna Japonica.

[40] Hilzheimer H (1905). Uber einige Tigerschadel aus der Strassburger Zoologischen Sammlung.. Zoologischer Anzeiger.

[41] Schwarz E (1912). Notes on Malay tigers, with description of a new form from Bali.. Annals and Magazine of Natural History, Series 8.

[42] Pocock R (1929). Tigers.. Journal of the Bombay Natural History Society.

[43] Mazak V (1968). Nouvelle sous-espece de tigre provenant de l'Asie due Sud-Est.. Mammalia.

[44] Lopez JV, Cevario S, O'Brien SJ (1996). Complete nucleotide sequences of the domestic cat (Felis catus) mitochondrial genome and a transposed mtDNA tandem repeat (Numt) in the nuclear genome.. Genomics.

