# Supplementary material for: Mitochondrial Phylogeography Illuminates the Origin of the Extinct Caspian Tiger and Its Relationship to the Amur Tiger
Source: PLoS One. 2009 Jan 14;4(1):e4125. doi: 10.1371/journal.pone.0004125 (PMC2624500; doi:10.1371/journal.pone.0004125)
Supplement: Table S1 — Caspian tiger specimens studied (0.06 MB DOC) [file pone.0004125.s001.doc]

| Table S1 | Caspian tiger specimens studied |  |  |  |
| --- | --- | --- | --- | --- |
| LGD accession no. | Museum | Museum number | Collection notes on tiger origin | Date sampled |
| PTV-8 | Institute of Zoology, Almaty, Kazakhstan | 2/10974 | Kazalinsky, Kzylorda (near Aral Sea), Kazakhstan | 1933 |
| PTV-1 | Institute of Zoology, Almaty, Kazakhstan | 4/10995 | Seven Rivers, Almaty region, S.E. Kazakhstan | no date |
| PTV-98 | Institute of Zoology, Almaty, Kazakhstan | 1/10973/2 | Ili River, near Lake Balkhash, Kazakhstan | 1930s |
| PTV-7 | Medical College, Baku, Azerbaijan | left* | Lankaran, Azerbaijan | 1936-1942 |
| PTV-9 | Medical College, Baku, Azerbaijan | right* | Lankaran, Azerbaijan | 1936-1942 |
| PTV-6 | Zoological Museum, M.S.U., Moscow | s-33151 | Ili River, S.E. Kazakhstan | 1939 - not the date of kill |
| PTV-5 | Zoological Museum, M.S.U., Moscow | S-38285 | N. Persia - Moscow Zoo | 1942 |
| PTV-11 | Zoological Museum, M.S.U., Moscow | S-52871 | Piandji River, Pamir Mountains, Tajik-Afgan border | 13/03/1950 |
| PTV-10 | Zoological Museum, R.A.S., Novosibirsk | 7181(2226) | Kurgan-Tyubinskaya, Tadjikistan | 1/8/51 |
| PTV-4 | Zoological Museum, R.A.S., Novosibirsk | 7182(2225) | Kurgan-Tyubinskaya, Tadjikistan | 1944 |
| PTV-23 | Zoological Museum, R.A.S., St. Petersburg | 1512(1654) | Kitai, Lobnor, Xingiang, People's Republic of China | 1878 |
| PTV-21 | Zoological Museum, R.A.S., St. Petersburg | 4046 | Kitai, Kulvdja, Lobnor, Xingiang, People's Republic of China | 1888 |
| PTV-15 | Zoological Museum, R.A.S., St. Petersburg | 7862(2983) | Kitai, Lobnor, Xingiang, People's Republic of China | 1889 |
| PTV-99 | Zoological Museum, R.A.S., St. Petersburg | 5728 | Tashkent, Uzbekistan | 1873 |
| PTV-14 | Zoological Museum, R.A.S., St. Petersburg | 5730(3027) | Kirgiskie steppe, Kazakhstan | no date |
| PTV-24 | Zoological Museum, R.A.S., St. Petersburg | 5734 | Amu-Darya | no date |
| PTV-17 | Zoological Museum, R.A.S., St. Petersburg | 9391(8679) | Fergana, Uzbekistan | 1903 |
| PTV-19 | Zoological Museum, R.A.S., St. Petersburg | 9387 | Syr-darya, Kazapa (Kazaly?, near Aral Sea?) | no date |
| PTV-18 | Zoological Museum, R.A.S., St. Petersburg | 9386 | Pyandj (Panj: Tajik-Afgan border), Darkatz (?) | no date |
| PTV-3 | Zoological Museum, R.A.S., St. Petersburg | 9392(8678) | River Atrak, S.W. Turkmenistan | no date |
| PTV-22 | Zoological Museum, R.A.S., St. Petersburg | 14997 | Turkmenia | 1887 |
| PTV-20 | Zoological Museum, R.A.S., St. Petersburg | 33110 | Turkmenia | presented in 1989 |
| PTV-16 | Zoological Museum, R.A.S., St. Petersburg | 2981 | Turkestan (present Uzbekistan?) | 1877 |
| PTV-13 | Zoological Museum, R.A.S., St. Petersburg | 3030 | North coast of Balhash Lake, Kazakhstan | 1877 |
| PTV-2 | Zoological Museum, R.A.S., St. Petersburg | 1407 | Amu-Darya | no date |
| PTV-12 | Zoological Museum, R.A.S., St. Petersburg | 3032 | Tian Shan Mountains, Kazakhstan | 1879 |
|  |  |  |  |  |

* C.A.D. notes that these tigers were on display flanking the main stairway and have no discernable accession numbers.

Abbreviations as follows: PTV- *Panthera tigris virgata*; L. G. D.- Laboratory of Genomic Diversity; R. A. S. Russian Academy of Sciences; M. S. U.- Moscow State University
