# Supplementary material for: Mitochondrial Phylogeography Illuminates the Origin of the Extinct Caspian Tiger and Its Relationship to the Amur Tiger
Source: PLoS One. 2009 Jan 14;4(1):e4125. doi: 10.1371/journal.pone.0004125 (PMC2624500; doi:10.1371/journal.pone.0004125)
Supplement: Table S2 — P. t. virgata individuals PCR amplified and sequenced at each fragment (0.05 MB DOC) [file pone.0004125.s002.doc]

Table S2. ***P. t. virgata* individuals PCR amplified and sequenced at each fragment**

| Fragment | PCR Product | No. variable/diagnostic sites | *virgata* typic alleles | LGD Ptv ID# | N |
| --- | --- | --- | --- | --- | --- |
|  |  |  |  |  |  |
|  |  |  |  |  |  |
| ND5 | 96 | 1/1 | T | 2, 6, 7, 10, 17, 18 | 6 |
|  |  |  |  |  |  |
| ND6 | 209 | 6/3+ | T,C,A | 2, 3, 6, 10, 13, 15, ***17***, 18, 21, ***22, 23*** | 11 |
|  |  |  |  |  |  |
| CytB-a | 144 | 3/1 | T,G,G | 2, 3, 6, 10, 12, 13f, 17, 18, 22 | 9 |
|  |  |  |  |  |  |
| CytB-b | 119 | 3/1 | C,A,C | 2, 3, 6, 10, 13, 15, 18, 22, 23 | 9 |
|  |  |  |  |  |  |
| ND2-a | 192 | 2/2 | C,T | 1, 2, 3, 6, 9, 12, 17, 19, 99 | 9 |
|  |  |  |  |  |  |
| ND2-b | 137 | 4/3 | A,G,G,C | 2, 3, 4, 5, 6, 10, 12, 13, 17, 18, 21, 22 | 12 |
|  |  |  |  |  |  |
| ND2-c | 124 | 3/2 | T,G,C | 1, 2, 3, 6, 7, 11, 12, 13, 15*, 17, 18 | 11 |
|  |  |  |  |  |  |
| CO1 | 120 | 2/1 | T,G | 2, 12, 17, 21 | 4 |
|  |  |  |  |  |  |
|  |  |  |  |  |  |
| Total |  | 24/14 |  |  | 20 |

Boxed T is *virgata* SNP matching *altaica* diagnostic SNP. Underlined T is the single SNP at which *virgata* and *altaica* differ.  ***Bold italicized*** indicate autapomorphically variable individuals. f = forward sequence only. * individual with incomplete sequence, missing 1^st^ diagnostic SNP. +note that for ND6 three of the six variable sites are autapomorphic to *P. t. virgata*. Note also that this report raises the total number of variable sites at this segment to 24 from the 21 reported in Luo et al., 2004.
