# Supplementary material for: Mitochondrial Phylogeography Illuminates the Origin of the Extinct Caspian Tiger and Its Relationship to the Amur Tiger
Source: PLoS One. 2009 Jan 14;4(1):e4125. doi: 10.1371/journal.pone.0004125 (PMC2624500; doi:10.1371/journal.pone.0004125)
Supplement: Table S3 — Variable sites among tigers in an alignment of 4 kb of concatenated mtDNA sequences used in phylogenetic analysis. (0.22 MB DOC) [file pone.0004125.s003.doc]

| Table S3: Variable sites among tigers in an alignment of 4 kb of concatenated mtDNA sequences used in phylogenetic analysis.   |  | | | | | | | | | | | | | | | | | | | | | | | | | | | | | | | | | | | | | | | | | | | | | | | | | | | | | | | | | | --- | --- | --- | --- | --- | --- | --- | --- | --- | --- | --- | --- | --- | --- | --- | --- | --- | --- | --- | --- | --- | --- | --- | --- | --- | --- | --- | --- | --- | --- | --- | --- | --- | --- | --- | --- | --- | --- | --- | --- | --- | --- | --- | --- | --- | --- | --- | --- | --- | --- | --- | --- | --- | --- | --- | --- | --- | | Subspecies | | | | | | | | | | | | | | | | | | | | | | | | | | | | | | | | | | | | | | | | | | | | | | | | | | | | | | | | | | AMOHaplotype ID | AMO1 | A | C | G | C | C | G | C | A | C | T | C | C | C | T | C | C | G | C | T | T | T | G | T | G | G | C | A | T | C | T | C | T | A | C | A | T | G | A | T | G | C | C | A | T | C | A | A | G | C | C | A | C | C | T | G | |  | SUM1 | . | . | . | . | . | . | . | . | . | C | . | . | . | . | T | . | . | . | . | . | . | . | . | . | . | . | G | . | . | . | . | C | G | . | . | . | A | . | C | . | . | . | . | . | T | . | G | . | T | T | G | . | . | . | . | |  | SUM2 | . | . | . | . | . | . | . | . | . | C | . | . | . | . | T | . | . | . | . | . | . | . | . | . | . | . | G | . | . | . | . | . | . | . | . | . | A | . | C | . | . | . | . | . | T | . | G | . | T | T | G | . | . | . | . | |  | SUM3 | . | . | . | . | . | . | . | . | . | C | . | . | . | . | T | . | . | . | . | . | . | . | C | . | . | . | G | . | . | . | . | . | . | . | . | . | A | . | C | . | . | . | . | . | T | . | G | . | T | T | G | . | . | . | . | | SUM | SUM4 | G | . | . | . | . | . | . | . | . | C | . | . | . | . | T | . | . | . | . | . | . | . | . | . | . | . | G | . | . | . | . | . | G | . | . | . | A | . | C | . | . | . | . | . | T | . | G | . | T | T | G | . | . | . | . | |  | SUM5 | . | . | . | . | . | . | . | . | . | C | . | . | . | . | T | . | . | . | . | C | . | . | . | . | . | . | G | . | . | . | . | . | . | . | . | . | A | . | C | . | . | . | . | . | T | . | G | . | T | T | G | . | . | . | . | |  | SUM6 | . | . | . | . | . | . | . | . | . | C | . | . | . | . | T | . | . | . | C | . | . | . | . | . | . | . | G | . | . | . | . | . | . | . | . | . | A | . | C | . | . | . | . | . | T | . | G | . | T | T | G | . | . | . | A | |  | SUM7 | . | . | . | . | . | . | . | . | . | C | . | . | . | . | T | . | . | . | C | . | . | . | . | . | . | . | G | . | . | . | . | . | . | . | . | . | A | G | C | . | . | . | . | . | T | . | G | . | T | T | G | . | . | . | A | |  | SUM8 | . | . | . | . | . | . | . | . | T | C | . | T | T | . | T | . | . | . | C | . | . | . | . | . | . | . | G | . | . | . | . | . | . | . | . | . | A | G | C | . | . | . | . | . | T | . | G | . | T | T | G | . | . | . | A | |  | TIG1 | G | . | . | . | T | . | . | . | . | C | T | . | . | C | T | T | . | . | . | C | . | . | . | . | . | . | . | . | . | . | . | . | . | . | . | . | A | . | . | A | T | . | . | . | T | . | G | A | . | T | G | T | . | . | . | |  | TIG2 | G | . | . | . | . | A | . | . | . | C | . | . | . | C | T | T | . | . | . | C | . | . | . | . | . | . | . | . | . | . | . | . | . | . | . | . | A | . | C | . | T | . | . | . | T | . | G | A | . | T | G | . | . | . | . | | TIG | TIG3 | G | . | . | . | . | . | . | . | . | C | . | . | . | C | T | T | . | . | . | . | . | . | . | . | . | . | . | . | . | . | . | . | . | . | . | . | A | . | C | . | T | . | . | . | T | . | G | A | . | T | G | . | . | . | . | |  | TIG4 | G | . | . | . | . | . | . | . | . | C | . | . | . | C | T | T | . | . | . | C | . | . | . | . | . | . | . | . | . | . | . | . | . | T | . | . | A | . | C | . | T | . | . | . | T | . | G | A | . | T | G | . | . | . | . | |  | TIG5 | G | . | . | . | . | . | . | . | . | C | . | . | . | C | T | T | . | . | . | C | . | . | . | . | . | . | . | . | . | . | . | . | . | . | . | . | A | . | C | . | T | . | . | . | T | . | G | A | . | T | G | . | . | . | . | |  | TIG6 | G | . | . | . | . | . | . | . | . | C | . | . | . | C | T | T | . | . | . | C | . | . | . | . | . | . | . | . | . | . | . | . | . | . | . | . | A | . | C | . | T | . | . | . | T | . | G | A | . | T | G | T | . | . | . | |  | COR4 | G | . | A | . | . | . | . | . | . | C | . | . | . | C | T | . | . | . | . | . | . | A | . | A | . | . | . | C | T | . | . | . | . | T | . | . | A | . | . | . | . | T | . | . | T | . | G | . | . | T | G | . | . | . | . | | COR | COR1 | G | T | A | . | . | . | . | . | . | C | . | . | . | C | T | . | . | . | . | . | . | A | . | A | . | . | . | C | T | . | . | . | . | T | . | . | A | . | . | . | . | T | . | . | T | . | G | . | . | T | G | . | . | . | . | |  | COR2 | G | T | A | . | . | . | . | . | . | C | . | . | . | C | T | . | . | . | . | . | . | A | . | A | . | . | . | C | T | . | . | . | . | T | . | . | A | . | . | . | . | T | G | . | T | . | G | . | . | T | G | . | . | . | . | |  | COR3 | G | T | A | . | . | . | . | . | . | C | . | . | . | C | T | . | . | . | . | . | . | A | . | A | . | . | . | C | T | . | . | . | . | T | . | C | A | . | . | . | . | T | . | . | T | . | G | . | . | T | G | . | . | . | . | |  | JAX1 | G | . | . | . | . | . | . | . | . | C | . | . | . | C | T | . | . | . | . | . | . | . | . | . | A | T | . | . | . | . | T | . | . | . | G | . | A | . | . | . | . | T | . | . | T | . | G | . | . | T | G | . | . | . | . | |  | JAX2 | G | . | . | . | . | . | . | . | . | C | . | . | . | C | T | . | . | . | . | . | . | . | . | . | . | T | . | . | . | . | T | . | . | . | G | . | A | . | . | . | . | T | . | . | T | . | G | . | . | T | G | . | . | . | . | | JAX | JAX3 | G | . | . | T | . | . | . | T | . | C | . | . | . | C | T | . | . | . | . | . | . | . | . | . | . | . | . | . | . | C | . | . | . | . | . | . | A | . | . | . | . | T | . | . | T | G | G | . | . | T | G | . | . | . | . | |  | JAX5 | G | . | . | T | . | . | . | T | . | C | . | . | . | C | T | . | . | T | . | . | . | . | . | . | . | . | . | . | . | C | . | . | . | . | . | . | A | . | . | . | . | T | . | . | T | G | G | . | . | T | G | . | . | . | . | |  | JAX4 | G | . | . | . | . | . | . | T | . | C | . | . | . | C | T | . | . | T | . | . | C | . | . | . | . | . | . | . | . | C | . | . | . | . | . | . | A | . | . | . | . | T | . | . | T | G | G | . | . | T | G | . | . | . | . | | ALT | ALT | G | . | A | . | . | . | T | . | . | C | . | . | . | C | T | . | A | . | . | . | . | . | . | . | . | . | . | C | . | . | . | . | . | T | . | . | A | . | . | . | . | T | . | C | T | . | G | . | . | T | G | . | . | C | . | | VIR | PTV-2 | - | - | - | - | - | - | T | - | - | - | - | - | - | - | T | . | A | - | - | - | - | - | . | . | . | . | . | C | - | - | - | - | - | - | - | - | - | - | - | - | - | - | - | C | T | . | G | . | . | T | G | . | C/T | . | . | |  |  |  |  |  |  |  |  |  |  |  |  |  |  |  |  |  |  |  |  |  |  |  |  |  |  |  |  |  |  |  |  |  |  |  |  |  |  |  |  |  |  |  |  |  |  |  |  |  |  |  |  |  |  |  |  |  | |
| --- | --- | --- | --- | --- | --- | --- | --- | --- | --- | --- | --- | --- | --- | --- | --- | --- | --- | --- | --- | --- | --- | --- | --- | --- | --- | --- | --- | --- | --- | --- | --- | --- | --- | --- | --- | --- | --- | --- | --- | --- | --- | --- | --- | --- | --- | --- | --- | --- | --- | --- | --- | --- | --- | --- | --- | --- | --- | --- | --- | --- | --- | --- | --- | --- | --- | --- | --- | --- | --- | --- | --- | --- | --- | --- | --- | --- | --- | --- | --- | --- | --- | --- | --- | --- | --- | --- | --- | --- | --- | --- | --- | --- | --- | --- | --- | --- | --- | --- | --- | --- | --- | --- | --- | --- | --- | --- | --- | --- | --- | --- | --- | --- | --- | --- | --- | --- | --- | --- | --- | --- | --- | --- | --- | --- | --- | --- | --- | --- | --- | --- | --- | --- | --- | --- | --- | --- | --- | --- | --- | --- | --- | --- | --- | --- | --- | --- | --- | --- | --- | --- | --- | --- | --- | --- | --- | --- | --- | --- | --- | --- | --- | --- | --- | --- | --- | --- | --- | --- | --- | --- | --- | --- | --- | --- | --- | --- | --- | --- | --- | --- | --- | --- | --- | --- | --- | --- | --- | --- | --- | --- | --- | --- | --- | --- | --- | --- | --- | --- | --- | --- | --- | --- | --- | --- | --- | --- | --- | --- | --- | --- | --- | --- | --- | --- | --- | --- | --- | --- | --- | --- | --- | --- | --- | --- | --- | --- | --- | --- | --- | --- | --- | --- | --- | --- | --- | --- | --- | --- | --- | --- | --- | --- | --- | --- | --- | --- | --- | --- | --- | --- | --- | --- | --- | --- | --- | --- | --- | --- | --- | --- | --- | --- | --- | --- | --- | --- | --- | --- | --- | --- | --- | --- | --- | --- | --- | --- | --- | --- | --- | --- | --- | --- | --- | --- | --- | --- | --- | --- | --- | --- | --- | --- | --- | --- | --- | --- | --- | --- | --- | --- | --- | --- | --- | --- | --- | --- | --- | --- | --- | --- | --- | --- | --- | --- | --- | --- | --- | --- | --- | --- | --- | --- | --- | --- | --- | --- | --- | --- | --- | --- | --- | --- | --- | --- | --- | --- | --- | --- | --- | --- | --- | --- | --- | --- | --- | --- | --- | --- | --- | --- | --- | --- | --- | --- | --- | --- | --- | --- | --- | --- | --- | --- | --- | --- | --- | --- | --- | --- | --- | --- | --- | --- | --- | --- | --- | --- | --- | --- | --- | --- | --- | --- | --- | --- | --- | --- | --- | --- | --- | --- | --- | --- | --- | --- | --- | --- | --- | --- | --- | --- | --- | --- | --- | --- | --- | --- | --- | --- | --- | --- | --- | --- | --- | --- | --- | --- | --- | --- | --- | --- | --- | --- | --- | --- | --- | --- | --- | --- | --- | --- | --- | --- | --- | --- | --- | --- | --- | --- | --- | --- | --- | --- | --- | --- | --- | --- | --- | --- | --- | --- | --- | --- | --- | --- | --- | --- | --- | --- | --- | --- | --- | --- | --- | --- | --- | --- | --- | --- | --- | --- | --- | --- | --- | --- | --- | --- | --- | --- | --- | --- | --- | --- | --- | --- | --- | --- | --- | --- | --- | --- | --- | --- | --- | --- | --- | --- | --- | --- | --- | --- | --- | --- | --- | --- | --- | --- | --- | --- | --- | --- | --- | --- | --- | --- | --- | --- | --- | --- | --- | --- | --- | --- | --- | --- | --- | --- | --- | --- | --- | --- | --- | --- | --- | --- | --- | --- | --- | --- | --- | --- | --- | --- | --- | --- | --- | --- | --- | --- | --- | --- | --- | --- | --- | --- | --- | --- | --- | --- | --- | --- | --- | --- | --- | --- | --- | --- | --- | --- | --- | --- | --- | --- | --- | --- | --- | --- | --- | --- | --- | --- | --- | --- | --- | --- | --- | --- | --- | --- | --- | --- | --- | --- | --- | --- | --- | --- | --- | --- | --- | --- | --- | --- | --- | --- | --- | --- | --- | --- | --- | --- | --- | --- | --- | --- | --- | --- | --- | --- | --- | --- | --- | --- | --- | --- | --- | --- | --- | --- | --- | --- | --- | --- | --- | --- | --- | --- | --- | --- | --- | --- | --- | --- | --- | --- | --- | --- | --- | --- | --- | --- | --- | --- | --- | --- | --- | --- | --- | --- | --- | --- | --- | --- | --- | --- | --- | --- | --- | --- | --- | --- | --- | --- | --- | --- | --- | --- | --- | --- | --- | --- | --- | --- | --- | --- | --- | --- | --- | --- | --- | --- | --- | --- | --- | --- | --- | --- | --- | --- | --- | --- | --- | --- | --- | --- | --- | --- | --- | --- | --- | --- | --- | --- | --- | --- | --- | --- | --- | --- | --- | --- | --- | --- | --- | --- | --- | --- | --- | --- | --- | --- | --- | --- | --- | --- | --- | --- | --- | --- | --- | --- | --- | --- | --- | --- | --- | --- | --- | --- | --- | --- | --- | --- | --- | --- | --- | --- | --- | --- | --- | --- | --- | --- | --- | --- | --- | --- | --- | --- | --- | --- | --- | --- | --- | --- | --- | --- | --- | --- | --- | --- | --- | --- | --- | --- | --- | --- | --- | --- | --- | --- | --- | --- | --- | --- | --- | --- | --- | --- | --- | --- | --- | --- | --- | --- | --- | --- | --- | --- | --- | --- | --- | --- | --- | --- | --- | --- | --- | --- | --- | --- | --- | --- | --- | --- | --- | --- | --- | --- | --- | --- | --- | --- | --- | --- | --- | --- | --- | --- | --- | --- | --- | --- | --- | --- | --- | --- | --- | --- | --- | --- | --- | --- | --- | --- | --- | --- | --- | --- | --- | --- | --- | --- | --- | --- | --- | --- | --- | --- | --- | --- | --- | --- | --- | --- | --- | --- | --- | --- | --- | --- | --- | --- | --- | --- | --- | --- | --- | --- | --- | --- | --- | --- | --- | --- | --- | --- | --- | --- | --- | --- | --- | --- | --- | --- | --- | --- | --- | --- | --- | --- | --- | --- | --- | --- | --- | --- | --- | --- | --- | --- | --- | --- | --- | --- | --- | --- | --- | --- | --- | --- | --- | --- | --- | --- | --- | --- | --- | --- | --- | --- | --- | --- | --- | --- | --- | --- | --- | --- | --- | --- | --- | --- | --- | --- | --- | --- | --- | --- | --- | --- | --- | --- | --- | --- | --- | --- | --- | --- | --- | --- | --- | --- | --- | --- | --- | --- | --- | --- | --- | --- | --- | --- | --- | --- | --- | --- | --- | --- | --- | --- | --- | --- | --- | --- | --- | --- | --- | --- | --- | --- | --- | --- | --- | --- | --- | --- | --- | --- | --- | --- | --- | --- | --- | --- | --- | --- | --- | --- | --- | --- | --- | --- | --- | --- | --- | --- | --- | --- | --- | --- | --- | --- | --- | --- | --- | --- | --- | --- | --- | --- | --- | --- | --- | --- | --- | --- | --- | --- | --- | --- | --- | --- | --- | --- | --- | --- | --- | --- | --- | --- | --- | --- | --- | --- | --- | --- | --- | --- | --- | --- | --- | --- | --- | --- | --- | --- | --- | --- | --- | --- | --- | --- | --- | --- | --- | --- | --- | --- | --- | --- | --- | --- | --- | --- | --- | --- | --- | --- | --- | --- | --- | --- | --- | --- | --- | --- | --- | --- | --- | --- | --- | --- | --- | --- | --- | --- | --- | --- | --- | --- | --- | --- | --- | --- | --- | --- | --- | --- | --- | --- | --- | --- | --- | --- | --- | --- | --- | --- | --- | --- | --- | --- | --- | --- | --- | --- | --- | --- | --- | --- | --- | --- | --- | --- | --- | --- | --- | --- | --- | --- | --- | --- | --- | --- | --- | --- | --- | --- | --- | --- | --- | --- | --- | --- | --- | --- | --- | --- | --- | --- | --- | --- | --- | --- | --- | --- | --- | --- | --- | --- | --- | --- | --- | --- | --- | --- | --- | --- | --- | --- | --- | --- | --- | --- | --- | --- | --- | --- | --- | --- | --- | --- | --- | --- | --- | --- | --- | --- | --- | --- | --- | --- | --- | --- | --- | --- | --- | --- | --- | --- | --- | --- | --- | --- | --- | --- | --- | --- | --- | --- | --- | --- | --- | --- | --- | --- | --- | --- | --- | --- | --- | --- | --- | --- | --- | --- | --- | --- | --- | --- | --- | --- | --- | --- | --- | --- | --- | --- | --- | --- | --- | --- | --- | --- | --- | --- | --- | --- | --- | --- | --- | --- | --- | --- | --- | --- | --- | --- | --- | --- | --- | --- | --- | --- | --- | --- | --- | --- | --- | --- | --- | --- | --- | --- | --- | --- | --- | --- | --- | --- | --- | --- | --- | --- | --- | --- | --- | --- | --- | --- | --- | --- | --- | --- | --- | --- | --- | --- | --- | --- | --- | --- | --- | --- | --- | --- | --- | --- | --- | --- | --- | --- | --- | --- | --- | --- | --- | --- | --- | --- | --- | --- | --- | --- | --- | --- | --- | --- | --- | --- | --- | --- | --- | --- | --- | --- | --- | --- | --- | --- | --- | --- | --- | --- | --- | --- | --- | --- | --- | --- | --- | --- | --- | --- | --- | --- | --- | --- | --- | --- | --- | --- | --- | --- | --- | --- | --- | --- | --- | --- | --- | --- | --- | --- | --- | --- | --- | --- | --- | --- | --- | --- | --- | --- | --- | --- | --- | --- | --- | --- | --- | --- | --- | --- | --- | --- | --- | --- | --- | --- | --- | --- | --- | --- | --- | --- | --- | --- | --- | --- | --- | --- | --- | --- | --- | --- | --- | --- | --- | --- | --- | --- | --- | --- | --- | --- | --- | --- | --- | --- | --- | --- | --- | --- | --- | --- | --- | --- | --- | --- | --- | --- | --- | --- | --- | --- | --- | --- | --- | --- | --- | --- | --- | --- | --- | --- | --- | --- | --- | --- | --- | --- | --- | --- | --- | --- | --- | --- | --- | --- | --- | --- | --- | --- | --- | --- | --- | --- | --- | --- | --- | --- | --- | --- | --- | --- | --- | --- | --- | --- | --- | --- | --- | --- | --- | --- | --- | --- | --- | --- | --- | --- | --- | --- | --- | --- | --- | --- | --- | --- | --- | --- | --- | --- | --- | --- | --- | --- | --- | --- | --- | --- | --- | --- | --- | --- | --- | --- | --- | --- | --- | --- | --- | --- | --- | --- | --- | --- | --- | --- | --- | --- | --- | --- | --- | --- | --- | --- | --- | --- | --- | --- | --- | --- | --- | --- | --- | --- | --- | --- | --- | --- | --- | --- | --- | --- | --- | --- | --- | --- | --- | --- | --- | --- | --- | --- | --- | --- | --- | --- | --- | --- | --- | --- | --- | --- | --- | --- | --- | --- | --- | --- | --- | --- | --- | --- | --- | --- | --- | --- | --- | --- | --- | --- | --- | --- | --- | --- | --- | --- | --- | --- | --- | --- | --- | --- | --- | --- | --- | --- | --- | --- | --- |
| Boxes indicate three synapomorphic sites common to Caspian and Amur tigers. Underlined is a single derived nucleotide found in all Amur (ALT) tigers but not in other subspecies. Points indicate matches to the reference AMO1 sequence. Shading indicates less-informative regions not sequenced in Caspian tigers. Tiger subspecies are abbreviated as follows: ALT, *Panthera tigris altaica*, Amur or "Siberian" tiger; AMO, *P. t. amoyensis*, South China tiger; COR, *P. t. corbetti*, Indochinese tiger; JAX, *P. t. jacksoni*, Malayan tigers; SUM, *P. t. sumatrae*, Sumatran tiger; TIG, *P. t. tigris*, Bengal tiger. Sequence except PTV-2 from Luo et al., 2004. The alignment excludes the clouded leopard (*Neofelis nebulosa*) sequence used used to root the tree, Genbank DQ257669. Ptv-2 was a Caspian tiger specimen for which all sequencing attempted on Caspian tigers, *P. t. virgata*, was successful. Ptv-2 also carried the most common Caspian tiger haplotype, although one nucleotide in Ptv-2 ("C/T") appeared to be heteroplasmic. A few Caspian tiger haplotypes each differed from Ptv-2 by a single nucleotide difference; each a derived state not found among any other tigers. |
